# Fluid intelligence but not need for cognition is associated with attitude change in response to the correction of misinformation

**DOI:** 10.1186/s41235-024-00595-1

**Published:** 2024-09-18

**Authors:** Fabian Hutmacher, Markus Appel, Benjamin Schätzlein, Christoph Mengelkamp

**Affiliations:** Human-Computer-Media-Institute, Psychology of Communication and New Media, Julius-Maximilians-University Würzburg, Oswald-Külpe-Weg 82, 97074 Würzburg, Germany

**Keywords:** Misinformation, Fluid intelligence, Propositional reasoning, Need for cognition, Continued influence effect

## Abstract

Misinformation can profoundly impact an individual’s attitudes—sometimes even after the misinformation has been corrected. In two preregistered experiments (*N*_1_ = 355, *N*_2_ = 725), we investigated whether individual differences in the ability and motivation to process information thoroughly influence the impact of misinformation in a news media context. More specifically, we tested whether fluid intelligence and need for cognition predicted the degree to which individuals who were exposed to misinformation changed their attitudes after receiving a correction message. We found consistent evidence that higher fluid intelligence is associated with a more pronounced correction effect, while need for cognition did not have a significant effect. This suggests that integrating a correction message with a previously encountered piece of misinformation can be challenging and that correction messages consequently need to be communicated in a way that is accessible to a broad audience.

**Supplementary Information:**

The online version contains supplementary material available at 10.1186/s41235-024-00595-1.

## Significance statement

The spread of misinformation can be considered one of the key challenges for contemporary societies. A common strategy for countering misinformation is correcting and fact-checking false information after they have been published (debunking). In research on debunking, it has repeatedly been observed that a correction message diminishes but not always fully eliminates the effects of previously presented misinformation. We investigated whether the extent to which individuals rely on misinformation despite receiving a correction message varies depending on their general reasoning ability (fluid intelligence) as well as their willingness to use this ability and to engage in effortful thinking (need for cognition). Across two studies, we found consistent evidence that individuals with higher fluid intelligence change their attitudes more in response to a correction message, while need for cognition did not have a significant effect. From a practical perspective, our results suggest that it is particularly important to communicate correction messages in a way that is accessible to a broad audience; that is, media outlets interested in successful debunking need to take into account that integrating a correction message with a previously encountered piece of misinformation can be challenging for individuals.

## Introduction

The spread of misinformation can be considered one of the key challenges for contemporary societies (for an overview, see, e.g., Ecker et al., 2022; van der Linden, 2022): Although misinformation has already existed in the pre-digital age, the recent technological developments allow creating and sharing misinformation in an unprecedented manner, which could ultimately undermine the rational public discourse and the formation of unbiased opinions. A common strategy for countering misinformation is correcting and fact-checking false information after they have been published (*debunking*). In research on debunking (for a meta-analysis, see Chan et al., 2017), it has repeatedly been observed that a correction message diminishes (*correction effect*) but does not fully eliminate the effects of previously presented misinformation, a phenomenon known as the *continued influence effect* (CIE; for a meta-analysis, see Walter & Tukachinsky, 2020; for reviews, see Ecker et al., 2022; Lewandowsky et al., 2012). The extent to which individuals rely on misinformation despite a correction varies, with rather stable individual differences assumed to play a substantial role (e.g., Brydges et al., 2018; Susmann & Wegener, 2022). Our focus here is on the influence of individual differences in the ability and motivation to process information thoroughly. More specifically, we investigated whether fluid intelligence and the willingness to show these abilities (need for cognition) predict the degree to which individuals who have been exposed to misinformation change their attitudes after receiving a correction message. Exploring individual differences in this context is not only interesting from a theoretical perspective but could also have important practical implications: In case correction messages should turn out to be less effective for certain groups of individuals, this would provide a clear rationale for developing debunking strategies especially targeted at these groups.

### Investigating the continued impact of misinformation

In the standard laboratory paradigm for investigating the continued impact of misinformation, participants are assigned to one of two conditions (for the classic studies, see Johnson & Seifert, 1994; Wilkes & Leatherbarrow, 1988; see also Seifert, 2002): the experimental group is presented with material on a certain topic that contains some misinformation; later, participants in the experimental group read a correction message, informing the participants about the incorrect piece of information. The control group is also presented with material on the same topic. However, this material does not contain any misinformation and is hence not followed by a correction message. Typically, despite the correction, the experimental group is still influenced by the misinformation and does not attain the same level of correctness (e.g., Brydges et al., 2018) or the same level of attitudes (e.g., De keersmaecker & Roets, 2017) as the control group. In the present experiments, we decided to focus on the topic of trust-based working time, because we assumed that participants would neither have sophisticated knowledge nor strong opinions with respect to trust-based working time. The basic idea behind trust-based working time is that workers are allowed to organize their working time independently and on their own responsibility (for more details, see the materials). We hypothesized that we would replicate the two effects known from previous studies, namely that the correction message reduces (correction effect) but not eliminates (continued influence effect) the impact of the misinformation on the individuals’ attitudes:

- *H1-Correction Effect*: In the experimental group, the attitude towards trust-based working time is more positive after reading the correction message than directly after reading the news text including misinformation.
- *H2-Continued Influence Effect (CIE)*: Even after reading the correction message, participants in the experimental group will have a more negative attitude towards trust-based working time than participants in the control group.

The correction effect and the CIE are closely related from a conceptual point of view: The more pronounced the correction effect, the smaller the continued influence effect. More specifically, the more individuals in the experimental group adjust their attitudes in response to receiving a correction message, the smaller the difference between experimental group and control group. Nevertheless, the two effects can help to answer different questions. Even if there is no difference regarding attitude between experimental group and control group after reading the correction message (i.e., if there is no significant CIE), for instance, it is still possible that the degree to which participants in the experimental group change their attitude in response to reading a correction message is influenced by individual differences.

### Understanding the continued impact of misinformation: cognitive mechanisms and individual differences

Broadly speaking, there are two different accounts for explaining the mechanisms underlying the continued impact of misinformation, which both draw on assumptions about the functioning of human memory: the *integration account* and the *selective retrieval account* (cf. Ecker et al., 2022). Proponents of the integration account postulate that for a correction message to be effective, the misinformation, which has been encoded first, needs to be co-activated and integrated with the correction message (e.g., Ecker et al., 2017; Ithisuphalap et al., 2020; Kendeou et al., 2014). In case individuals do not detect a conflict between the misinformation and the correction message and/or in case they do not understand that the correction message invalidates the originally presented misinformation, the misinformation will continue to influence the individuals’ attitudes and behavior. Proponents of the selective retrieval account hold that the continued impact of misinformation stems from the fact that the misinformation can be retrieved from memory without also retrieving the correction message, while retrieving the correction message will automatically also trigger the retrieval of the misinformation (e.g., Ecker et al., 2011; Gordon et al., 2019). This asymmetry makes the misinformation appear more familiar than the correction message, which may lead individuals to assume that the misinformation is still valid. Importantly, these two accounts are not mutually exclusive: Based on electrophysiological evidence, it has been suggested that the continued impact of misinformation is driven by the combination of a strong representation of the misinformation in memory followed by the failure to integrate the correction message with the misinformation (Brydges et al., 2020).

Apart from these general cognitive mechanisms, the degree to which misinformation continues to have an effect on individuals even after receiving a correction message may depend on a range of individual differences. For instance, it has been observed that being presented with a correction message can produce feelings of psychological discomfort and that individuals who perceive more psychological discomfort are more susceptible to the continued impact of misinformation (Susmann & Wegener, 2022). In addition, the impact of a piece of misinformation as well as the effectiveness of a correction message may crucially depend on how credible and trustworthy individuals find the information that they are being presented with (e.g., Ecker & Antonio, 2021; Guillory & Geraci, 2013; Vraga & Bode, 2018). In line with this, the effectiveness of a correction message also depends on an individual’s worldview in the sense that a correction message that is perceived as a threat to one’s values and identity can be ineffective (e.g., Ecker & Ang, 2019; Nyhan & Reifler, 2010).

Following in the footsteps of such an individual differences perspective, we decided to investigate two cognitive parameters that might play a role in understanding the continued impact of misinformation. As outlined in detail below, we suggest that fluid intelligence, the domain-independent reasoning ability, will influence how well individuals are able to integrate a correction message with a piece of misinformation. In addition, the continued impact of misinformation might also be shaped by the individuals’ willingness to use these abilities and to engage in effortful thinking (i.e., need for cognition).

#### Fluid intelligence

One of the most widely used and most widely cited theories of intelligence is the Cattell–Horn–Carroll theory, which recognizes intelligence as multidimensional, with a general factor at the highest stratum and multiple group factors at a lower stratum (Carroll, 1993; for an overview, see McGrew, 2009; Schneider & McGrew, 2018). One of these group factors, which is particularly highly correlated with general intelligence, is fluid intelligence: It “can be defined as the use of deliberate and controlled procedures […] to solve novel, ‘on-the-spot’ problems that cannot be solved by using previously learned habits, schemas, and scripts” (Schneider & McGrew, 2018, p. 93). More specifically, fluid intelligence is considered to include the ability for inductive and deductive reasoning as well as the ability for quantitative (mathematical) reasoning. In the context of the continued impact of misinformation, we consider the ability for deductive reasoning to be particularly important, as deductive reasoning involves using logical principles to draw conclusions from given premises (cf. Gühne et al., 2021), and as this is precisely what individuals need to do when deducing from a correction message that the correction message invalidates the previously presented misinformation, that is, when *integrating* the available information. Hence, we decided to measure deductive reasoning as an important facet of fluid intelligence; moreover, as we used verbal stimulus material, the items that we selected for measuring deductive reasoning were also verbal in nature (for details, see Methods).

To our knowledge, there are no previous studies investigating the influence of fluid intelligence on the continued impact of misinformation in the context of news media content, that is, in the context in which misinformation arguably plays the biggest role in contemporary societies (cf. Lazer et al., 2018). However, one study investigated the influence of intelligence on the continued impact of misinformation in the context of social evaluations (De keersmaecker & Roets, 2017). Participants read a description of a young woman that contained misinformation about her stealing drugs, and this misinformation was corrected afterward. The misinformation had a stronger continued impact on individuals with lower levels of intelligence, whereas participants with higher levels of intelligence were no longer influenced by the misinformation after receiving a correction message. However, intelligence in this study was measured using a subtest from the Wechsler Adult Intelligence Scale (WAIS) requiring participants to select the best synonym to a target word, that is, a subtest measuring crystallized rather than fluid intelligence. On a more general note, it has been observed that forming false memories based on misinformation is negatively associated with intelligence (Zhu et al., 2010).

Apart from that, there are several studies investigating the relationship between the continued impact of misinformation and working memory capacity, finding that higher working memory capacity is associated with a reduced impact of misinformation after receiving a correction message (Brydges et al., 2018; Jia et al., 2020; McIlhiney et al., 2023; Wenjuan et al., 2023; but see Sanderson et al., 2021). This is particularly interesting, as fluid intelligence and working memory capacity are usually considered to be connected albeit theoretically distinct concepts, although the nature of their relationship is not yet fully understood. While some argue that being able to maintain and update information is an important precondition for reasoning, it has also been suggested to view working memory and fluid intelligence as complementary processes (for an overview, see, e.g., Burgoyne et al., 2019; Shipstead et al., 2016). In any case, the findings regarding working memory capacity lend further credibility to our hypothesis:

- *H3—Fluid Intelligence*: The size of the correction effect is influenced by fluid intelligence. More specifically, higher fluid intelligence leads to a more pronounced correction effect.

#### Need for Cognition

Even if individuals have the necessary cognitive abilities to process and integrate a correction message with a previously presented piece of misinformation, they may still choose to go with their gut feelings and intuitions instead of actively and systematically engaging with the available evidence (cf. Pennycook & Rand, 2019, 2021; Rudloff & Appel, 2022; Rudloff et al., 2022). One way of conceptualizing this individual disposition is in terms of one’s *need for cognition* (NFC). The NFC denotes “the tendency for an individual to engage in and enjoy thinking” (Cacioppo & Petty, 1982, p. 116). Individuals with a high NFC prefer complex and intellectually challenging to simple problems, while individuals with a low NFC tend to avoid tasks that require deliberation and mental effort (for an overview, see Cacioppo et al., 1996; Petty et al., 2009). Direct evidence for the role of NFC in the context of the continued impact of misinformation is sparse: In one of the above-mentioned studies (De keersmaecker & Roets, 2017), including a related construct (need for closure) as a control variable did not change the results. In another study (Vafeiadis & Xiao, 2021), NFC influenced the individuals’ behavioral intentions after being informed about a piece of misinformation, their judgments of the quality of the correction, and their engagement with the correction (i.e., their willingness to like, share, and comment on the correction) but not their attitude towards the topic. Thus, the study provides some evidence for NFC influencing the processing of the correction. Based on this sparse evidence, we decided to formulate two open research questions with respect to NFC. First, we were interested whether or not NFC predicts the size of the correction effect (RQ1). Second, we were interested in the influence of NFC on the postulated effects of fluid intelligence. Does the postulated effect of fluid intelligence hold once NFC is controlled for (RQ2)? Moreover, we inspected a possible higher-order interaction between fluid intelligence and NFC (RQ3). Finally, we examined moderation effects of fluid intelligence and NFC on the CIE in a post hoc analysis.

## Experiment 1

### Method

Our first experiment was conducted online via Qualtrics in a single-factor between-subjects design, in which one group (experimental group) received misinformation that was corrected afterwards, whereas another group (control group) did not receive any misinformation. Attitude towards the focal topic was measured twice, both in the control group and in the experimental group: the first time after the information phase and the second time after both groups had completed the items on fluid intelligence and the experimental group had read the correction. Fluid intelligence and need for cognition served as continuous predictors of the correction effect (i.e., the difference between attitude before and after reading the correction message). We report all manipulations, measures, and exclusions in our experiments. The material is available in Online Supplement S4.

#### Participants

Participants were recruited via posts on various websites, including Facebook and SurveyCircle, and via the university’s participant recruitment system. As an incentive, participants could either participate in a lottery to win 25€ or received course credit. Based on the study by De keersmaecker and Roets (2017) who sampled 390 participants, we aimed for a sample size of 400 participants who completed the experiment and met the German language requirement. In total, 401 participants completed the experiment. As preregistered, we excluded participants who did not answer both control questions correctly (*n* = 6), did not pass the attention check (*n* = 10), reported taking notes during the fluid intelligence test (*n* = 5), reported low diligence (*n* = 7), took less than ten seconds to read at least one of the stimulus texts (*n* = 10), completed the reasoning test in less than two minutes (*n* = 6) or completed the experiment in less than four minutes (*n* = 2). Our final sample consisted of 355 participants (17–67 years, *M* = 26.6, SD = 10.6, 104 male, 249 female, and 2 nonbinary). Most participants were university students (for more details about sample demographics, see Online Supplement S1).

#### Material

Our focal topic was a fictitious pilot project in which workers in a tech company in Estonia could organize their own working time (see Online Supplement S4 for the entire material). In the control condition a newspaper article was presented, describing a pilot project in which employees were allowed to decide about their working time on their own without any restrictions. The text stated that the pilot project was aimed at creating more family-friendly and more attractive working conditions and that the Estonian government hoped that fewer employees would emigrate to Western European countries in the long run. No information was given about the results of the pilot project (neutral text, 143 words). In the experimental condition, a text was presented in which the pilot project was portrayed as a failure; that is, the neutral text was expanded with information about financial losses for the company as a result of employees working late in the evening and therefore being less efficient (misinformation text, 268 words). After a first set of dependent and predictor variables was assessed (see Procedure), participants in the experimental group read a debunking message from a (fictitious) independent fact-checking website stating that the misinformation text was based on a lack of journalistic care and reporting an additional statement by an expert who draws a more nuanced picture on the effects of introducing trust-based working time; that is, the misinformation text was depicted as erroneous. Following the suggestion by Lewandowsky et al. (2012) to fill possible gaps in the mental model created by the debunking message, the new information was provided that the financial losses were the result of expired supply contracts of the company, implying that the pilot project may have been more successful than suggested in the misinformation text (correction text, 182 words).

#### Instruments

##### Fluid intelligence

Propositional reasoning can be used as a valid indicator of fluid intelligence (cf. Gühne et al., 2021; Schneider & McGrew, 2018). The propositional reasoning test proposed by Gühne et al. (2021) consists of 15 item families. Each item family includes a certain set of premises (e.g., “If P or Q, then R”, “P”) and five possible conclusions from which only one is correct (e.g., “R”). For each of these item families, one can create a large item pool using automatic item generation based on R code made available through the authors of the test (see The International Cognitive Ability Resource Team, 2014). For the above-mentioned item family, a sample item includes the premises “If the oil drips or the engine whirrs, then the pedal is depressed” and “The oil is dripping,” with the correct conclusion being that “The pedal is depressed.” For the present study, we selected ten of these 15 item families to keep the study at a reasonable length. Given the reliability of the propositional reasoning test (Gühne et al., 2021, Study 3), we expected to achieve acceptable reliability levels even with fewer items. For each of the ten selected item families, five items were generated, out of which one randomly selected item was shown to each participant so that each participant responded to ten items. To estimate internal consistency, we calculated Revelle’s omega because Cronbach’s alpha is underestimating the internal consistency when tau equivalence (i.e., equal item variance, equal standardized factor loadings) is violated (Dunn et al., 2014; McNeish, 2017). As recommended by McNeish (2017), we used polychoric covariance matrices instead of Pearson covariance matrices to calculate the internal consistency of fluid intelligence. We used the psych-package Version 2.4.1 in R Version 4.3.2 for the calculation of Revelle’s omega. The internal consistency (i.e., Revelle’s omega total) was 0.89.

##### Need for cognition

Need for cognition was measured using a four-item short-scale (Beißert et al., 2015; e.g., “I would prefer more complicated problems over simple problems”). Items were answered on a 7-point rating scale from 1 *(strongly disagree)* to 7 *(strongly agree)*. Revelle’s omega total was 0.70.

##### Attitude

The attitude towards trust-based working time was measured using eight items, two each targeting the cognitive (e.g., “I think that trust-based working time can work”), affective (e.g., “When I think about trust-based working time, I have a good feeling”), conative (e.g., “I would support the introduction of trust-based working time at as many workplaces as possible”), and evaluative (e.g., “I believe that trust-based working time is a good thing”) component of attitude towards trusted working time (see Online Supplement S4 for a list of all items). The items were presented in randomized order for each participant. Each item was answered on a 7-point rating scale from 1 *(strongly disagree)* to 7 *(totally agree)*. Revelle’s omega total was 0.92 both before and after reading the news article.

#### Procedure

After providing informed consent, participants indicated their knowledge of German and their age. Then, participants were instructed to read the following texts thoroughly. In the experimental group, the misinformation text was shown, while participants in the control group read the neutral text. Following the reading task, participants had to answer a control question about the name of the company that was mentioned in the text. Afterwards, they filled in the items about their attitude towards trust-based working time (*t*1). This was followed by the propositional reasoning test: First, the task format was explained, supported by two practice questions including elaborative feedback; then, the ten items were shown one by one. Participants were instructed to tick their best guess if they were not sure and to refrain from taking notes (which was checked through a control item after completing the propositional reasoning test). Then participants in the experimental group read the correction text, followed again by a control question asking for the name of the expert that was cited in the correction text, whereas those in the control group did not read the correction text. Next, the attitude items had to be filled in a second time, both in the experimental group and in the control group (*t*2, including one attention check item). Subsequently, participants answered the NFC items and responded to demographic questions regarding gender, education, and occupation, and a question if they had participated in the experiment diligently. Finally, participants were debriefed about the fictional character of the stimulus texts. In total, the study lasted *Mdn* = 16 min.

## Results

Note that a Bonferroni correction was preregistered for Experiment 1. Given that previous studies on individual differences in the context of the continued impact of misinformation did usually not adjust their alpha level (e.g., Brydges et al., 2018; De keersmaecker & Roets, 2017), we discarded the Bonferroni correction for better comparability in favor of a more standard alpha level (i.e., Type I error was set to alpha = 0.05). As preregistered, we detected outliers using box plots (i.e., values exceeding 1.5 times the interquartile range from Q1 and Q3) and adjusted the outliers to the least non-outlying value ± one unit (for the statistical background, see Field, 2018, p. 264; Tabachnick & Fidell, 2013, p. 111). We adjusted 7 values for the attitude at *t*1, 7 values at *t*2, and 3 values in fluid intelligence; for NFC no outliers were detected. Means and standard deviations for all variables are shown in Online Supplement S2, and correlations are shown in Online Supplement S3. We additionally calculated all analyses without adjustments to check for the effect of adjusting outlying values (see Online Supplement S5). All significances concerning hypotheses and research questions remained the same.

To evaluate the manipulation check and test the hypotheses on the correction effect (H1) and the CIE (H2), mixed ANOVAs are calculated as preregistered, with the attitude after reading the newspaper article at *t*1 and the attitude after the correction at *t*2. The simple effect between the groups at *t*1 tests whether the incorrect information affected the attitude which can be considered as a manipulation check. The simple effect between the groups at *t*2 tests whether a CIE is present, and the simple effect of time for the experimental group tests whether the correction was successful (i.e., correction effect).

**Fig. 1:**
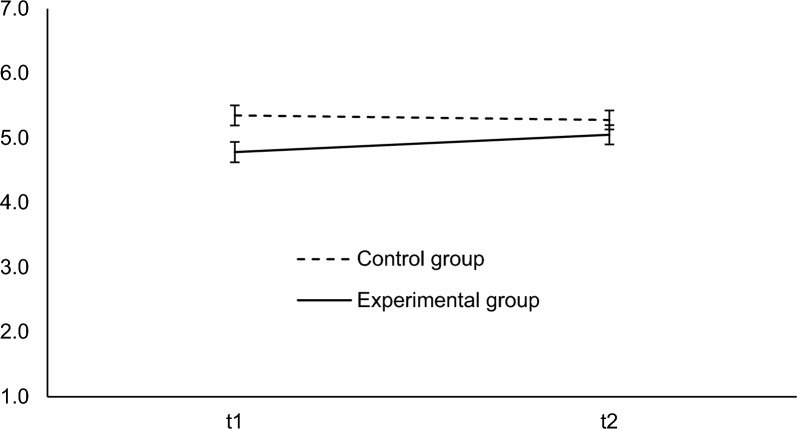
Attitude at *t*1 and *t*2 in Experiment 1. Attitude from 1 (*strongly disagree*) to 7 (*totally agree*)*.* Error bars indicate the 95% confidence interval

We found a significant main effect of group, *F*(1, 353) = 13.98, *p* < 0.001, ${\eta }_{p}^{2}$ = 0.04, a significant main effect of time, *F*(1, 353) = 15.22, *p* < 0.001, ${\eta }_{p}^{2}$ = 0.04, and a significant interaction effect of group and time,* F*(1, 353) = 44.97, *p* < 0.001, ${\eta }_{p}^{2}$ = 0.11. The results are depicted in Fig. 1. Simple effect analyses revealed that the misinformed group had a lower attitude towards trusted working hours at *t*1 compared to the control group, *F*(1, 353) = 25.60, *p* < 0.001, *d* = 0.54, indicating that the misinformation had an effect (manipulation check). Further, the groups did differ significantly after the correction was presented, *F*(1, 353) = 4.60, *p* = 0.033, *d* = 0.23, i.e., we found a small CIE (*d*-values were calculated using Lenhard & Lenhard, 2022). Moreover, there was a significant increase in attitude from *t*1 to *t*2 for the experimental group, *F*(1, 353) = 55.47, *p* < 0.001, ${\eta }_{p}^{2}$ = 0.14, *d*_*RM*_ = 0.51, i.e., the correction had an effect. In the control group, the attitude did significantly decrease from *t*1 to *t*2 albeit to a small extent, *F*(1, 353) = 3.99, *p* = 0.047, *d*_*RM*_ = − 0.17.

We calculated a hierarchical regression analysis to test if the correction effect was influenced by fluid intelligence (H3) and by NFC (RQs). Note that in the preregistration (for both Experiment 1 and Experiment 2), we had stated that we would run independent regression analyses for the two predictors (i.e., fluid intelligence and NFC). Upon closer consideration, however, it seemed more reasonable from both a theoretical and a methodological perspective to investigate the impact of the two predictors on attitude change (i.e., the difference between the attitude *t*2 minus *t*1) in one hierarchical regression analysis. Model 2 (see below) equals the preregistered regression analysis for fluid intelligence. For NFC, running the preregistered analyses does not change the results in a meaningful way (see Supplement S6). In our analysis, the simple slope of fluid intelligence on attitude change in the experimental group tests whether fluid intelligence can predict the correction effect (H3). Furthermore, the simple slope of NFC on attitude change in the experimental group shows whether NFC affected the correction effect over and above the effect of fluid intelligence (RQ1). Finally, the interaction of fluid intelligence and NFC as a predictor of attitude change in the experimental group shows whether the effect of fluid intelligence on attitude change is moderated by NFC (RQ2). Simple slopes in the control group are of no interest for testing these hypotheses and research questions but provide a control for effects of fluid intelligence and NFC on the stability of the attitude.

In model 1, we used group (dummy-coded using the control group as the reference category) and fluid intelligence (*M* = 0.77, SD = 0.16) as the predictors and attitude change as the dependent variable. The predictors explained *R*^2^ = 0.12 of variance, *F*(2, 352) = 22.95, *p* < 0.001, and group significantly predicted attitude change (confirming our ANOVA results) whereas fluid intelligence did not (see Table 1 for coefficients of all regression models). Next, we added the interaction effect group x fluid intelligence to the regression (model 2). The model did not improve significantly, Δ*R*^2^ = 0.01, *F*(1, 351) = 3.45, *p* = 0.064. However, as the interaction effect was close to significant and the simple slope for the experimental group reflects the effects of fluid intelligence as hypothesized in H3, we calculated a simple slope analysis. In support of H3, we observed a significant simple slope of fluid intelligence for the experimental group, *b* = 0.46, *β* = 0.15, *p* = 0.044, 95% CI [0.01, 0.29], but not for the control group, *b* = − 0.11, *β* = − 0.04, *p* = 0.585, 95% CI [− 0.17, 0.10].

**Table 1:** Regression of attitude change on experimental group, fluid intelligence, and NFC

Variable	Model 1		Model 2		Model 3		Model 4		Model 5		Model 6	
	b	SE	b	SE	b	SE	b	SE	b	SE	b	SE
Experiment 1												
Group	.34***	.05	− .10***	.24	− .09***	.24	.04***	.32	.04***	.32	.68***	1.08
Fluid intelligence	.15###	.15	− .11***	.21	− .13***	.21	− .14***	.21	.53***	.70	.97***	1.00
Group × fluid intelligence			.57***	.31	.57***	.31	.59***	.31	.59***	.31	− .24***	1.38
NFC					.02***	.03	.01***	.03	.13***	.12	.06***	.16
Group × NFC							.03***	.05	.03***	.05	.18***	.24
Fluid intelligence × NFC									− .15***	.15	− .06***	.21
Group × fluid intelligence × NFC											− .19***	.30
*R*^2^	.12***		.12***		.13***		.13***		.13***		.13***	
Δ*R*^2^			.01***		< .01***		< .01***		< .01***		< .01***	
Experiment 2												
Group	.25***	.03	− .20***	.20	− .20***	.20	− .11***	.24	− .08***	.24	− 1.41***	1.02
Fluid intelligence	.27***	.11	.02***	.16	.03***	.16	.02***	.16	1.38***	.62	.62***	.84
Group × fluid intelligence			.53***	.23	.53***	.23	.55***	.23	.54***	.23	2.12***	1.21
NFC					− .02***	.02	− .01***	.02	.23***	.11	.10***	.15
Group × NFC							− .02***	.03	− .03***	.03	.25***	.21
Fluid intelligence × NFC									− .28***	.12	− .12***	.17
Group × fluid intelligence × NFC											− .33***	.25
*R*^2^	.10***		.10***		.10***		.11***		.11***		.11***	
Δ*R*^2^			.01***		.01***		< .01***		.01***		< .01***	

Group was dummy-coded using the control group as the reference

***p < .001

To answer our first research question (RQ1) as to whether NFC predicts the correction effect, we added NFC to our regression (model 3), which did not improve the model significantly, Δ*R*^2^ < 0.01, *F*(1, 350) = 0.94, *p* = 0.334. Adding the interaction group x NFC (model 4) did also not significantly increase the explained variance in attitude change, *F*(1, 349) = 0.43, *p* = 0.514, and therefore no evidence for NFC influencing the correction effect was found. Neither including the interaction fluid intelligence x NFC (model 5; RQ2), Δ*R*^2^ < 0.01, *F*(1, 348) = 1.00, *p* = 0.318, nor including the second-order interaction group x fluid intelligence x NFC (model 6; RQ3), Δ*R*^2^ < 0.01, *F*(1, 347) = 0.38, *p* = 0.537, improved the regression model any further.

Finally, we examined moderation effects on the CIE (i.e., on the difference in attitudes towards trust-based working time between the experimental group and the control group at *t*2). In this post hoc analysis, we calculated a regression in which we did not take the attitude at *t*1 into account (see De keersmaecker & Roets, 2017, for a similar analysis). First, we checked if we found a CIE when controlling for fluid intelligence, i.e., predictors were group (dummy-coded with the control group as the reference group) and fluid intelligence, attitude at *t*2 was the criterion. In this analysis, *R*^2^ = 0.02% of the variance in attitude at *t*2 was explained, *F*(2, 352) = 2.81, *p* = 0.062, and the group predicted attitude at *t*2 significantly, *b* = -0.23, *p* = 0.031, confirming the CIE effect that we have reported above. Fluid intelligence was no significant predictor of attitude at *t*2, *b* = − 0.33, *β* = − 0.05, *p* = 0.315. Next, we added the interaction group x fluid intelligence to the regression analysis. The predictors explained *R*^2^ = 0.02 of variance, *F*(3, 351) = 1.95, *p* = 0.121. However, the interaction effect was not significant, i.e., variance explained in attitude at *t*2 did not increase significantly, Δ*R*^2^ < 0.01, *F*(1, 351) = 0.26, *p* = 0.610. Therefore, fluid intelligence did not moderate the CIE if the attitude at *t*1 was not taken into account.

## Discussion

Experiment 1 was designed to investigate the effect of fluid intelligence and NFC on the continued impact of misinformation. To begin with, reading the text containing misinformation led to more negative attitudes towards trust-based working time in the experimental group than in the control group at *t*1. As hypothesized (H1), presenting the correction message led to a significant correction effect: In the experimental group, the attitude towards trust-based working time was more positive after reading the correction message than directly after reading the news text including misinformation. In addition, we found a significant CIE (H2): The attitude towards trust-based working time differed significantly between the experimental group and the control group after reading the correction message.

As hypothesized, we also found some support that higher fluid intelligence led to a more pronounced correction effect (H3). With respect to NFC, we did not find any evidence that NFC influences the size of the correction effect (RQ1). In addition, we found no evidence for higher-order interaction effects (RQ2). In sum, the results suggest that fluid intelligence plays a role in predicting the size of the correction effect, while there is no evidence for a significant influence of NFC. We decided to replicate and extend the experiment using a longer and hence arguably more reliable instrument for measuring NFC (i.e., the established standard 16-item NFC scale instead of a 4-item short measure) and the same items from the propositional reasoning test for all participants (instead of using variations of items from the same item families). In addition, we recruited a larger, more diverse online sample to account for the fact that most participants in Experiment 1 were university students.

## Experiment 2

### Method

#### Participants

Participants were recruited via Prolific (www.prolific.co) and compensated with £2.75. Hypothesis 3 (i.e., the hypothesis that fluid intelligence predicts the size of the correction effect) was our main hypothesis in Experiment 2. Therefore, we based our calculation of the sample size on the prediction of attitude change from fluid intelligence in Experiment 1 (partial *R*^2^ = 0.008614). We used G*Power (Faul et al., 2009) to calculate the required sample size of 713 participants (power 0.80, type I error 0.05, 3 predictors, one-tailed test). To account for potential exclusions, we aimed for a sample size of 790 participants who completed the experiment and met the German language requirement. In total, 795 participants completed the experiment. As preregistered, we excluded participants who did not fill in the check item correctly (*n* = 29), did not pass the attention check (*n* = 9), reported taking notes during the fluid intelligence test (*n* = 5), reported low diligence (*n* = 3), took less than ten seconds to read at least one of the stimulus texts (*n* = 10) or completed the reasoning test in less than two minutes (*n* = 14). Our final sample consisted of 725 participants (18–73 years, *M* = 30.6, SD = 9.7, 384 male, 325 female, 15 nonbinary, 1 missing). Most of the participants (*n* = 384) were employees, followed by students (*n* = 240), job-seekers (*n* = 43), apprentices (*n* = 28), and participants who still visited a school (*n* = 7). The remaining 23 participants reported other occupations. For more details about sample characteristics, see Online Supplement S1.

#### Materials and instruments

Materials were largely the same as in Experiment 1. We made slight adjustments regarding the measurement of NFC and fluid intelligence. NFC was measured using the German 16-item NFC scale (Bless et al., 1994). Items were answered on a 7-point rating scale from 1 *(strongly disagree)* to 7 *(strongly agree)*. Revelle’s omega total was 0.93. Fluid intelligence was measured using the same ten item families from the propositional reasoning test (Gühne et al., 2021) as in Experiment 1. However, we decided to present one version from each item family only and selected the item that worked best in Experiment 1 according to an item analysis. Revelle’s omega total was 0.85 for these ten items. Revelle’s omega total for the attitude scale was 0.94 both at *t*1 and at *t*2.

#### Procedure

The procedure was the same as in Experiment 1. In total, the study lasted *Mdn* = 14 min.

## Results

As preregistered, Type I error was set to 0.05 for all analyses, and we detected outliers using box plots (i.e., values exceeding 1.5 times the interquartile range from Q1 and Q3) and adjusted them to the least non-outlying value ± one unit (for the statistical background, see Field, 2018, p. 264; Tabachnick & Fidell, 2013, p. 111). We adjusted 18 values for the attitude at *t*1, 14 values for the attitude at *t*2, 8 values for NFC, and 86 values for fluid intelligence. All outliers with respect to fluid intelligence were outliers with low values in fluid intelligence. As performing items testing fluid intelligence is exhausting and challenging, it seems plausible that some participants guessed the answer instead of actually performing the task (at least on some trials), leading to 12% outliers. Therefore, it is reasonable to assume that the actual fluid intelligence of these participants was higher than their outlying value, and adjusting these values therefore reflects the true values better than the outlying values. In addition, we decided to calculate all analyses again without adjusting values in order to check the effect of the adjustments (cf. McClelland, 2000). The adjustments did not change the results (i.e., all significances remained the same, see Online Supplement S5). Means and standard deviations for all variables are shown in Online Supplement S2, correlations are shown in Online Supplement S3.

**Fig. 2:**
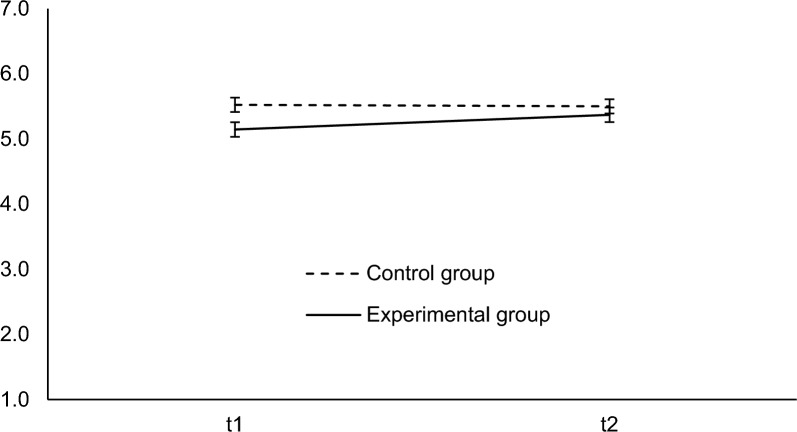
Attitude at *t*1 and *t*2 in Experiment 2. Attitude from 1 (*strongly disagree*) to 7 (*totally agree*)*.* Error bars indicate the 95% confidence interval

As in Experiment 1, we calculated a 2 (group) × 2 (time) mixed ANOVA to test the correction effect (H1) and the CIE (H2) using attitude as the dependent variable. Results showed a significant main effect of group,* F*(1, 723) = 10.46, *p* = 0.001, ${\eta }_{p}^{2}$ = 0.01, a significant main effect of the time, *F*(1, 723) = 46.19, *p* < 0.001, ${\eta }_{p}^{2}$ = 0.06, and a significant interaction effect, *F*(1, 723) = 70.49, *p* < 0.001, ${\eta }_{p}^{2}$ = 0.09. Simple effects analysis showed a significant effect of time for the experimental group, *F*(1, 723) = 113.06, *p* < 0.001, *d*_RM_ = 0.49, but not for the control group, *F*(1, 723) = 1.31, *p* = 0.254, *d*_RM_ = 0.07. Hence, we found support for a correction effect in the experimental group. The simple effect analyses rendered that group had a significant effect at *t*1, i.e., after reading the news but before the correction, *F*(1, 723) = 22.33, *p* < 0.001, *d* = 0.35, but no significant effect after the correction at *t*2, *F*(1, 723) = 2.65, *p* = 0.104, *d* = 0.13. Thus, the misinformation influenced the attitude at *t*1 (manipulation check), but we did not find evidence of a significant overall CIE after the correction. Results are plotted in Fig. 2.

To test if fluid intelligence predicted the correction effect (H3) and to answer our research questions about the effect of NFC (RQ1–RQ3), we calculated a hierarchical regression analysis. We used group and fluid intelligence as the predictors and attitude change as the dependent variable (model 1). In total, *R*^2^ = 0.10 variance in attitude change was explained, *F*(3, 722) = 38.25, *p* < 0.001. Group and fluid intelligence did both predict attitude change significantly (see Table 1 for regression coefficients). Adding the interaction effect group x fluid intelligence to the regression (model 2) did improve the model significantly, Δ*R*^2^ = 0.01, *F*(1, 721) = 5.28, *p* = 0.022. A simple slope analysis resulted in a significant effect of fluid intelligence on attitude change for the experimental group, *b* = 0.54, *β* = 0.17, *p* = 001, 95% CI [0.07, 0.27]. For the control group, fluid intelligence did not predict attitude change, *b* = 0.02, *β* < 0.01, *p* = 0.922, 95% CI [− 0.09, 0.10]. In other words, the correction effect in the experimental group was strengthened by fluid intelligence. Adding NFC to the regression (model 3) did not improve the model any further, Δ*R*^2^ < 0.01, *F*(1, 720) = 1.61, *p* = 0.205, and adding the interaction effect group x NFC to the regression (model 4) did not significantly increase the explained variance either, Δ*R*^2^ < 0.01, *F*(1, 719) = 0.48, *p* = 0.490. Calculating a regression analysis as preregistered without fluid intelligence did not change this result (see Online Supplement S6). Thus, we found no support for NFC strengthening the correction effect (RQ1). However, the interaction effect fluid intelligence × NFC did significantly improve the regression (model 5; RQ2), Δ*R*^2^ < 0.01, *F*(1, 718) = 5.18, *p* = 0.023.

**Fig. 3:**
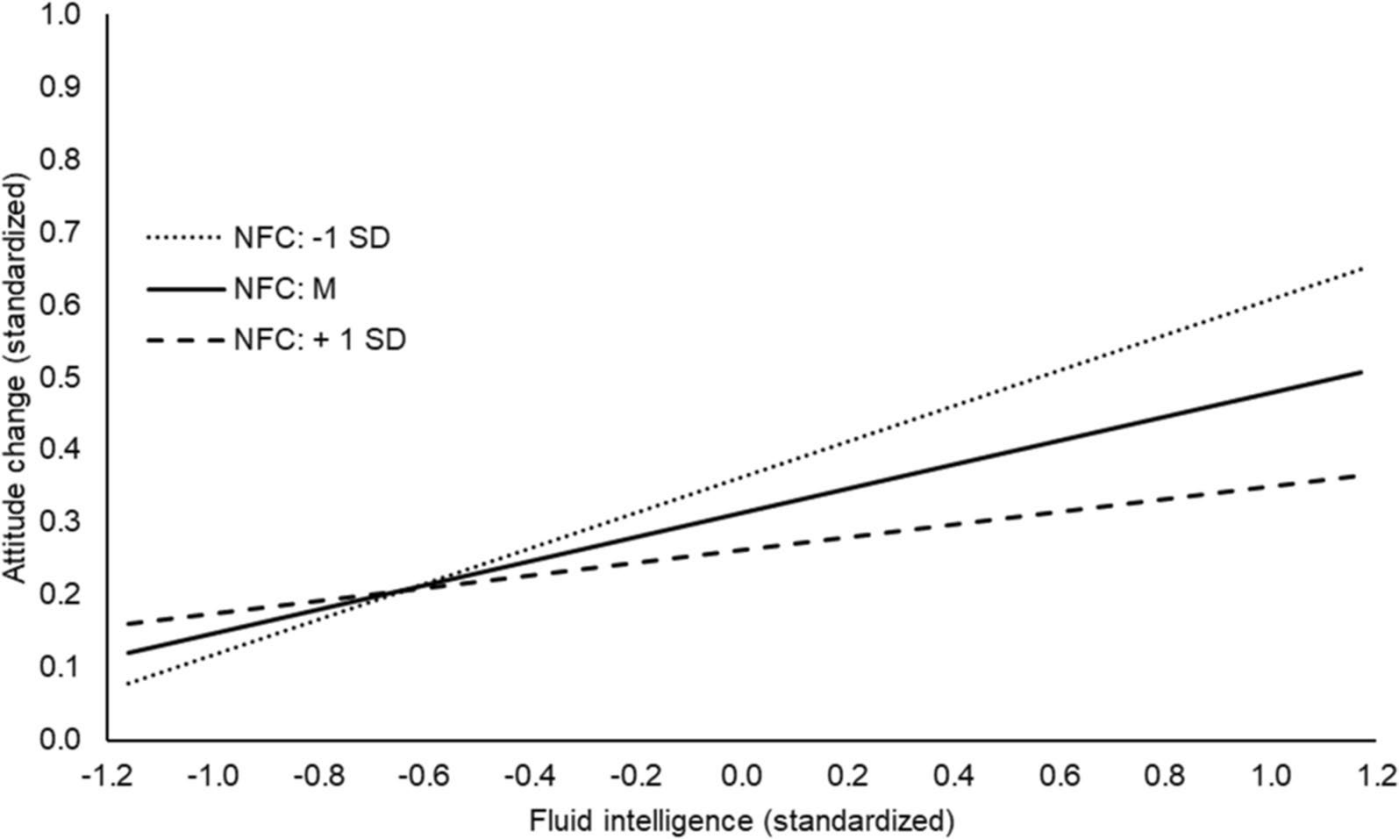
Effect of fluid intelligence on attitude change by NFC in the Experimental Group in Experiment 2. The figure depicts the effect of fluid intelligence on attitude change by NFC. Simple slopes for NFC 1 SD below average and for an average NFC are significantly different from zero

Figure 3 depicts the interaction effect of fluid intelligence by NFC for the experimental group (fluid intelligence, NFC, and attitude change were standardized for the sake of interpretation). For the experimental group, fluid intelligence had a significant effect on attitude change if NFC was one standard deviation below average, *β* = 0.24, *p* < 0.001, 95% CI [0.13, 0.36], and if NFC was average, *β* = 0.17, *p* = 0.001, 95% CI [0.07, 0.27]. However, the effect of fluid intelligence was no longer significant if NFC was one standard deviation above average, *β* = 0.09, *p* = 0.171, 95% CI [− 0.04, 0.21]. For the control group, fluid intelligence did not predict attitude change at any level of NFC (all *p*s > 0.153). To sum up, our post hoc analysis showed that the correction effect is predicted by fluid intelligence if NFC is low or average, i.e., the higher the fluid intelligence the stronger the correction effect. However, if NFC is high, fluid intelligence has no longer a significant effect on the correction effect. Finally, we checked if adding the second-order interaction group x fluid intelligence x NFC to the regression (model 6) did increase the explained variance in attitude change (RQ3). Model 6 did not fit the data significantly better compared to model 5, Δ*R*^2^ < 0.01, *F*(1, 717) = 1.79, *p* = 0.182.

We calculated the same post hoc regression analysis as in Experiment 1, i.e., we regressed the attitude at *t*2 on fluid intelligence and group without taking the attitude at *t*1 into account. In total, *R*^2^ < 0.01variance in attitude at *t*2 was explained, *F*(2, 722) = 1.35, *p* = 0.261. The results showed no significant main effect of the group, *b* = − 0.13, *p* = 0.104, and no significant main effect of fluid intelligence, *b* = 0.06, *p* = 0.844. Adding the interaction group x fluid intelligence to the regression model did not increase the variance explained in attitude at *t*2, Δ*R*^2^ < 0.01, *F*(1, 721) = 2.49, *p* = 0.115. Thus, the analysis provided no support of intelligence predicting the CIE if the attitude at *t*1 is not taken into account.

## General discussion

The present research was aimed at investigating whether fluid intelligence and NFC influence the continued impact of misinformation in a news media processing context. Across two experiments, we found fairly consistent evidence that fluid intelligence predicts the degree to which individuals who have been exposed to misinformation correct their attitudes after receiving a correction message in the sense that higher fluid intelligence is associated with a more pronounced correction effect, while NFC did not have a significant effect.

As far as fluid intelligence is concerned, these results confirm our hypothesis that higher fluid intelligence is helpful when having to integrate the content of a correction message with the content of a previously presented piece of misinformation. This finding is also in line with related previous research (De keersmaecker & Roets, 2017; Zhu, 2010). As already mentioned in the introduction, fluid intelligence is often considered to be connected to working memory capacity (for an overview, see, e.g., Burgoyne et al., 2019; Shipstead et al., 2016): On the one hand, being able to maintain and update information is sometimes seen as an important precondition for reasoning; on the other hand, it has also been argued that working memory and fluid intelligence are better understood as complimentary processes. While doing so goes way beyond the scope of the present paper, the fact that both higher fluid intelligence and higher working memory capacity are associated with a reduced impact of misinformation after receiving a correction message (Brydges et al., 2018; Jia et al., 2020; McIlhiney et al., 2023; Wenjuan et al., 2023; but see Sanderson et al., 2021) provides another reason why it would be important to disentangle the contributions of working memory and fluid intelligence to the processing of information in general and the processing of misinformation in particular. When conducting studies in this direction in the future, it might be worth considering the use of a more comprehensive measure of fluid intelligence. Although we chose the propositional reasoning test as a proxy for fluid intelligence based on theoretical grounds and although relying on a relatively short measure for fluid intelligence seemed appropriate to keep the workload manageable for participants, using measures that are able to capture the different sub-facets of fluid intelligence could help to gain deeper insights into the nature of our effects.

As far as the observation is concerned that NFC was unrelated to the size of the correction effect, our experiments confirm the preliminary evidence provided by related previous research (De keersmaecker & Roets, 2017; Vafeiadis & Xiao, 2021). As NFC denotes an individual’s tendency to engage in and enjoy thinking and as integrating a correction message with a previously presented piece of misinformation requires deliberating about the available evidence, this finding may nevertheless seem counterintuitive from a theoretical point of view. For instance, it has also been demonstrated that NFC is positively correlated with intellectual humility (e.g., Davis et al., 2016; Krumrei-Mancusi et al., 2020; Leary et al., 2017), a core component of which is being open to changing one’s mind. However, there is also research suggesting that individuals with a high NFC tend to have stronger and less ambivalent attitudes that are more resistant to change (Haugtvedt & Petty, 1992; Thompson & Zanna, 1995), arguably precisely because of their tendency to base their opinions on an effortful analysis of the available evidence (cf. Petty et al., 2009). In other words, it is possible that a higher NFC can also go hand in hand with more resistance to changing one’s attitude based on a correction message as individuals with a high NFC have invested considerable effort into building their attitude in the first place. Taken together, one explanation for our findings could be that the positive and negative effects of a high NFC are averaged out in our case. An alternative explanation could be that NFC is simply not related to correction effectiveness in a paradigm such as the one used in the present studies: More specifically, one may hypothesize that integrating the correction message with the previously presented piece of misinformation was relatively easy in the scenarios presented to the participants and that in order for NFC to play a significant role, the task would have to be more complex and challenging. As we see it, more research is needed to disentangle these different explanations. On a more general note, and although NFC is a well-accepted and widely used concept, one may also want to keep in mind that the standard measure of NFC is based on the participants’ self-assessment rather than on the participants’ performance on an actual task. Constructing a performance-based measure of NFC and connecting it to the performance in a paradigm such as the one used in the present studies could be an interesting avenue for future research (for the general background behind this idea, see Baumeister et al., 2007).

Interestingly, we observed an interaction between the influence of fluid intelligence and NFC on the size of the correction effect in Experiment 2 in the sense that fluid intelligence was only associated with a more pronounced correction effect at low and average levels of NFC, while fluid intelligence was unrelated to the correction effect at high levels of NFC. To be clear, this interaction effect was a post hoc finding that was not part of our original hypotheses. That being said, the direction of the interaction effect seems somewhat surprising: Intuitively, one might assume that—if anything—fluid intelligence should play a particular prominent role at high levels of NFC rather than at low levels of NFC. More specifically, it seems plausible to hypothesize that interindividual differences with respect to fluid intelligence should have a greater impact among participants who are motivated to think and to engage in difficult problems (i.e., who have a high NFC), and not so much among participants with a low NFC, that is, among participants who are less willing to use their cognitive abilities, even if they possess them. Given the lack of a convincing explanation for our post hoc finding and given that there was no evidence for an interaction effect in Experiment 1, we want to emphasize that our result should be treated with caution until it has been replicated in future research. In case it should turn out to be a replicable result, however, this would also create an urgent need for finding a plausible theoretical explanation.

Apart from that, there are at least two limitations to be noted. First, we decided to focus on the topic of trust-based working time, because we assumed that participants would neither have sophisticated knowledge nor strong opinions with respect to trust-based working time. From a practical perspective, however, reducing the effects of misinformation through debunking is arguably particularly relevant in the case of topics about which individuals do have prior knowledge and strong opinions (e.g., vaccination, climate change). Hence, it seems important to extend our findings to such more contested and more politicized topics. Second, we did not find a significant CIE in Experiment 2. That is, the correction message was so effective that there was no significant difference between the attitude towards trust-based working time between the experimental group and the control group after reading the correction message. Although the CIE is a well-established finding (for a meta-analysis, see Walter & Tukachinsky, 2020; for reviews, see Ecker et al., 2022; Lewandowsky et al., 2012), it has also been shown that correction messages can be particularly successful when they are detailed and when the topic under investigation is not highly politicized (cf. Chan & Albarracín, 2023; Chan et al., 2017). As both were the case in our experiments, it does not come as a surprise that the correction message that we provided in our experiments was quite effective. In addition, the way the different scenarios were designed in the present studies may have made finding a CIE less likely: The correction text presented in the experimental group did not merely state the misinformation text contained some piece of misinformation but corrected the misinformation by mentioning initial successes and increased employee satisfaction as a result of introducing trust-based working time. This positive evidence was not mentioned in the neutral text that the control group read at the beginning of the experiment, making it even more plausible that the attitudes between groups did not differ at the second measurement point. Note, however, that the fact that we did not find a significant CIE in Experiment 2 does not influence the validity of our main finding that higher fluid intelligence is associated with a more pronounced correction effect while NFC has no significant influence on the size of the correction effect. As already outlined in the introduction, even if there is no significant CIE, it is still possible that the degree to which participants in the experimental group change their attitude in response to reading a correction message is—as in our study—influenced by individual differences.

## Conclusion

Our findings do not only have theoretical but also practical implications: As lower fluid intelligence is associated with a less pronounced correction effect, it seems particularly important to communicate correction messages in a way that is accessible to a broad audience; that is, media outlets interested in successful debunking need to take into account that integrating a correction message with a previously encountered piece of misinformation can be challenging for individuals (for a similar line of thought in the context of motivated reasoning, see Hutmacher et al., 2022). Apart from these practical considerations, our present experiments contribute to understanding the individual differences underlying the continued impact of misinformation, that is, to understanding the factors that drive individuals to fall for misinformation even in the face of a correction message.

## Supplementary Information

Supplementary Material 1

## Data Availability

Data and code for both experiments can be accessed at 10.17605/OSF.IO/CDS8B. The material is provided in the Online Supplement. Both studies were preregistered (Study 1: https://aspredicted.org/fm4ii.pdf; Study 2: https://doi.org/10.17605/OSF.IO/RTJF2).
