# Supplementary material for: Fluid intelligence but not need for cognition is associated with attitude change in response to the correction of misinformation
Source: Cogn Res Princ Implic. 2024 Sep 18;9:64. doi: 10.1186/s41235-024-00595-1 (PMC11411052; doi:10.1186/s41235-024-00595-1)
Supplement: Supplementary file 1 — Supplementary Material 1 [file 41235_2024_595_MOESM1_ESM.docx]

# Online Supplement for

**“****Fluid Intelligence but not Need for Cognition is Associated with Attitude Change in Response to the Correction of Misinformation”**

S1 Demographics page 2

S2 Descriptive Statistics for Experiments 1 and 2 page 3

S3 Correlations for Experiments 1 and 2 page 4

S4 Material Experiments 1 and 2 page 5

S5 Results Without Adjusted Outliers page 11

S6 Preregistered Regression Analyses NFC page 17

# S1. Demographics Experiment 1 and 2

**Table S1**
*Demographics*

|  | Experiment 1 | | Experiment 2 | |
| --- | --- | --- | --- | --- |
|  | *n* | % | *n* | % |
| German language |  |  |  |  |
| Native speaker | 355 | 97.5 | 627 | 86.5 |
| Non native speaker | 9^a^ | 2.5 | 98^b^ | 13.5 |
| Education |  |  |  |  |
| Certificate of secondary ed. | 1 | 0.3 | 6 | 0.8 |
| General certificate of secondary ed. | 13 | 3.7 | 31 | 4.3 |
| A-levels | 219 | 61.7 | 230 | 31.7 |
| Completed vocational ed. | 31 | 8.7 | 85 | 11.7 |
| Graduate degree | 91 | 25.6 | 368 | 50.8 |
| Other | 0 | 0.0 | 3 | 0.4 |
| Occupation |  |  |  |  |
| Pupil | 4 | 1.1 | 7 | 1.0 |
| Student | 254 | 71.5 | 240 | 33.1 |
| Apprentice | 3 | 0.8 | 28 | 3.9 |
| Employed | 78 | 22.0 | 384 | 53.0 |
| Job-seeking / unemployed | 6 | 1.7 | 43 | 5.9 |
| Other | 10 | 2.8 | 23 | 3.2 |

*Note.*
^a^All 9 participants reported an excellent command of German.

^b^96 reported an excellent command of German.

# S2. Descriptive Statistics for Experiments 1 and 2

**Table S2**
*Descriptive Statistics*

| Group | N | Attitude | | | | Fluid Intelligence | | NFC | |
| --- | --- | --- | --- | --- | --- | --- | --- | --- | --- |
|  |  | t1 | | t2 | |  |  |  |  |
|  |  | *M* | *SD* | *M* | *SD* | *M* | *SD* | *M* | *SD* |
| Experiment 1 |  |  |  |  |  |  |  |  |  |
| Control | 180 | 5.35 | 0.92 | 5.28 | 0.97 | .78 | .17 | 4.56 | 0.91 |
| Experimental | 175 | 4.78 | 1.18 | 5.05 | 1.03 | .77 | .16 | 4.44 | 1.11 |
| Total | 355 | 5.07 | 1.09 | 5.17 | 1.01 | .77 | .16 | 4.50 | 1.02 |
| Experiment 2 |  |  |  |  |  |  |  |  |  |
| Control | 370 | 5.52 | 1.04 | 5.50 | 1.09 | .85 | .13 | 4.86 | 0.92 |
| Experimental | 355 | 5.14 | 1.12 | 5.37 | 1.06 | .85 | .13 | 4.85 | 0.93 |
| Total | 725 | 5.34 | 1.09 | 5.44 | 1.08 | .85 | .13 | 4.85 | 0.92 |

*Note.* Attitude from 1 *(strongly disagree)* to 7 *(totally agree)*, fluid intelligence as the proportion of correct responses, Need for Cognition (NFC) from 1 (*strongly disagree*) to 7 (*strongly agree*). All variables were winsorized.

# S3. Descriptive Statistics for Experiments 1 and 2

**Table S3**
*Correlations Across Conditions*

|  | 1 | 2 | 3 | 4 | 5 |
| --- | --- | --- | --- | --- | --- |
| 1. Fluid intelligence |  | **.12** | -.03 | < .01 | **.09** |
| 1. Need for cognition | .10 |  | **.16** | **.15** | -.04 |
| 1. Attitude t1 | -.07 | .03 |  | **.93** | **-.23** |
| 1. Attitude t2 | -.05 | .05 | **.89** |  | **.15** |
| 1. Attitude change | .04 | .03 | **-.39** | .08 |  |

*Note.*
Correlations for Experiment 1 (*N* = 355) were printed below the diagonal, and correlations for Experiment 2 (*N* = 725) were printed above the diagonal. Significant correlations (*p* < .05) are bolded. Note that correlations between attitudes and attitude change may be statistical artifacts (i.e., attitude change is derived from the attitudes at t1 and t2).

# S4. Material Experiments 1 and 2

**Text 1: Neutral Text**

***German version used in the studies***

Fauener Tagblatt, 3. März 2019

Testlauf zu eigenverantwortlicher Arbeitszeiteinteilung in Estland gestartet

Tallinn (fs). Das Modellprojekt zur eigenverantwortlichen Arbeitszeiteinteilung läuft nun seit einem Monat. Nach einer Ausschreibung des estländischen Ministeriums für Arbeit hatten sich drei Unternehmen, darunter das in der Hauptstadt ansässige Tech-Unternehmen Mindful, dafür entschieden, an dem Testlauf teilzunehmen. Wissenschaftler der Technischen Universität begleiteten das Experiment. […] Die Unternehmen stellen ihren Mitarbeitenden für einen Zeitraum von drei Monaten frei, wann und wie lange sie arbeiten. Wer zum Beispiel seine Aufgaben für den Tag in fünf Stunden schafft, kann entsprechend früher nach Hause gehen. Durch die eigenverantwortliche Einteilung der Arbeitszeit sollen die Unternehmen attraktiver und familienfreundlicher werden, so ein Sprecher des Ministeriums. Langfristig erhoffe man sich, dass die Zahl junger, gebildeter Estländer, die in westeuropäische Länder emigrieren, zurückgeht. […]

Der Test, an dem sich drei Unternehmen mit Sitz in Tallinn und Pärnu beteiligen, läuft noch bis Ende April.

***English version (created using DeepL)***

Fauener Tagblatt, March 3, 2019

Test run on self-responsible work time management launched in Estonia

Tallinn (fs). The pilot project on self-responsible work time management has now been running for a month. Following a call for tenders by the Estonian Ministry of Labor, three companies, including Mindful, a tech company based in the capital, had chosen to participate in the test run. Scientists from the Technical University accompanied the experiment. [...] The companies let their employees decide when and how long they work for a period of three months. For example, those who complete their tasks for the day in five hours can go home earlier. According to a spokesman of the ministry, the autonomous arrangement of working hours is intended to make companies more attractive and family-friendly. In the long term, it is hoped that the number of young, educated Estonians emigrating to Western European countries will decrease. [...]

The test, in which three companies based in Tallinn and Pärnu are participating, will run until the end of April.

**Text 2: Misinformation Text**

***German version used in the studies***

Fauener Tagblatt, 3. März 2019

Eigenverantwortliche Arbeitszeiteinteilung in Estland gescheitert

Tallinn (fs). Das Modellprojekt zur eigenverantwortlichen Arbeitszeiteinteilung läuft nun seit einem Monat. Nach einer Ausschreibung des estländischen Ministeriums für Arbeit hatten sich drei Unternehmen, darunter das in der Hauptstadt ansässige Tech-Unternehmen Mindful, dafür entschieden, an dem Testlauf teilzunehmen. Wissenschaftler der Technischen Universität begleiteten das Experiment. […] Die Unternehmen stellen ihren Mitarbeitenden für einen Zeitraum von drei Monaten frei, wann und wie lange sie arbeiten. Wer zum Beispiel seine Aufgaben für den Tag in fünf Stunden schafft, kann entsprechend früher nach Hause gehen. Durch die eigenverantwortliche Einteilung der Arbeitszeit sollen die Unternehmen attraktiver und familienfreundlicher werden, so ein Sprecher des Ministeriums. Langfristig erhoffe man sich, dass die Zahl junger, gebildeter Estländer, die in westeuropäische Länder emigrieren, zurückgeht.

Ants Tammsaare, Personalverantwortlicher für Mindful, zieht ein erstes Fazit: „Planvoll reduzierte Arbeitszeiten, wie wir sie in Schweden mit dem 6-Stunden-Tag gesehen haben, können Erfolg haben. Daher blickten wir sehr optimistisch auf unser Konzept. Zunächst wurde das Angebot auch von vielen unserer Mitarbeiterinnen und Mitarbeiter freudig angenommen. Allerdings mussten wir nach einem Monat Testlaufzeit feststellen, dass die Produktion um mehr als 25% gesunken ist“. So erlitt das Unternehmen einen Verlust von mehr als 300.000€. Die Gründe für den massiven Verlust sieht Tammsaare vor allem in den unregelmäßigen Zeiten, zu denen die Mitarbeitenden erschienen sind: „Unser Versuch, bei dem die Arbeitszeit komplett frei eingeteilt werden darf, führte dazu, dass einige Personen erst gegen 17:00 Uhr erschienen sind. Wenn man dann bis Mitternacht arbeitet, fällt die Konzentration stark ab.“ […]

Der Test, an dem sich insgesamt drei Unternehmen mit Sitz in Tallinn und Pärnu beteiligen, läuft noch bis Ende April.

***English version (created using DeepL)***

Fauener Tagblatt, March 3, 2019

Self-responsible work time allocation in Estonia failed

Tallinn (fs). The pilot project for self-responsible work time allocation has now been running for a month. Following a call for tenders by the Estonian Ministry of Labor, three companies, including Mindful, a tech company based in the capital, had chosen to participate in the trial run. Scientists from the University of Technology accompanied the experiment. [...] The companies let their employees decide when and how long they work for a period of three months. For example, those who complete their tasks for the day in five hours can go home earlier. According to a spokesman of the ministry, the autonomous arrangement of working hours is intended to make companies more attractive and family-friendly. In the long term, it is hoped that the number of young, educated Estonians emigrating to Western European countries will decrease.

Ants Tammsaare, HR Manager for Mindful, draws an initial conclusion: "Planned reduced working hours, as we have seen in Sweden with the 6-hour day, can be successful. So we were very optimistic about our concept. Initially, the offer was also happily accepted by many of our employees. However, after a month of testing, we found that production had dropped by more than 25%." Thus, the company suffered a loss of more than 300,000€. Tammsaare sees the reasons for the massive loss mainly in the irregular times at which employees showed up: "Our experiment, in which working hours are allowed to be completely freely arranged, resulted in some people not showing up until around 5:00 pm. If you then work until midnight, concentration drops sharply." [...]

The test, in which a total of three companies based in Tallinn and Pärnu are participating, will run until the end of April.

**Text 3: Correction Text**

***German version used in the studies***

Eine unabhängige Fact-Checking Website schreibt zu dem Bericht:

Bericht des Fauener Tagblatts unzureichend recherchiert

[…] Das Fauener Tagblatt berichtete in ihrer Freitagsausgabe vom 3. März 2019 über einen in Estland laufenden Test, bei dem Unternehmen ihren Mitarbeitenden eine freie Einteilung ihrer Arbeitszeit gewähren. Demnach kann sowohl die Uhrzeit als auch die Arbeitsdauer frei gewählt werden, solange alle Aufgaben erfüllt werden können. Im Titel des Artikels hieß es, der Testlauf sei „gescheitert“.

Wie eine weitere Recherche ergibt, bildet der Artikel die Situation des Unternehmens Mindful nicht umfangreich genug ab. Laut Wirtschaftsexperte Ernst Wengerer könne der massive Verlust des Technikunternehmens von 300.000€ durch abgelaufene Verträge mit zwei großen britischen Abnehmern erklärt werden. „Die neue Vertragssituation haben die entsprechenden Unternehmen nun auch in Pressemitteilungen bekannt gegeben“, so Wengerer. Außerdem glaube er nicht, dass die Konzentration der Mitarbeitenden abends wesentlich abfällt. […] Zunächst müsse man die Ergebnisse der Forschungsgruppe abwarten, die das Experiment begleitet.

Die zwei anderen Unternehmen, die sich am Testlauf beteiligen, konnten unterdessen erste Erfolge vermelden. Das Angebot werde internen Umfragen zufolge besonders von Eltern positiv wahrgenommen und habe die Zufriedenheit der Belegschaft steigern können. Der Umsatz der Unternehmen blieb dabei unverändert.

[…]

***English version (created using DeepL)***

An independent fact-checking website writes about the report:

Fauener Tagblatt report insufficiently researched

[...] In its Friday edition of March 3, 2019, the Fauener Tagblatt reported on a test underway in Estonia in which companies allow their employees to freely arrange their working hours. According to the report, both the time and duration of work can be freely chosen as long as all tasks can be completed. The title of the article stated that the test run had "failed."

As further research reveals, the article does not depict the situation of the Mindful company comprehensively enough. According to business expert Ernst Wengerer, the tech company's massive loss of €300,000 could be explained by expired contracts with two major British buyers. "The new contract situation has now also been announced by the respective companies in press releases," Wengerer said. In addition, he does not believe that the concentration of employees drops significantly in the evening. [...] Before drawing conclusions, one has to wait for the results of the research group that accompanies the experiment, he said.

Meanwhile, the two other companies participating in the test run reported initial successes. According to internal surveys, the offer is perceived positively, especially by parents, and has been able to increase employee satisfaction. The companies' sales remained unchanged.

[…]

**Attitude Measure: Items**

| No. | German (original) | English (translated) |
| --- | --- | --- |
| 1 | Ich denke, dass Vertrauensarbeitszeit funktionieren kann. | I think that trust-based working time can work. |
| 2 | Ich denke, wenn Arbeitszeiten nicht kontrolliert werden, nutzen Mitarbeitende das zu ihren Gunsten aus.* | I think that if working hours are not monitored, employees exploit this to their advantage.* |
| 3 | Ich würde mich dafür einsetzen, an möglichst vielen Arbeitsstellen Vertrauensarbeitszeit einzuführen. | I would support the introduction of trust-based working time at as many workplaces as possible. |
| 4 | Wenn sich ein Freund oder eine Freundin für eine eigenverantwortliche Arbeitszeiteinteilung ausspricht, würde ich dagegen argumentieren.* | If a friend argues in favor of trust-based working time, I would argue against it.* |
| 5 | Wenn ich an Vertrauensarbeitszeit denke, habe ich ein gutes Gefühl. | When I think about trust-based working time, I have a good feeling. |
| 6 | Beim Gedanken an Vertrauensarbeitszeit empfinde ich ein ungutes Gefühl.* | The thought of trust-based working time gives me a bad feeling.* |
| 7 | Vertrauensarbeitszeit halte ich für eine gute Sache. | I believe that trust-based working time is a good thing. |
| 8 | Ich finde, Vertrauensarbeitszeit ist eine schlechte Idee für die meisten Unternehmen.* | I think trust-based working time is a bad idea for most companies.* |

*Note.* Attitude from 1 (*strongly disagree*) to 7 (*totally agree*). *item reversed

# S5. Results Without Adjusted Outliers

To check for the effect of adjusting outliers, we report all descriptive and inferential results for Experiment 1 and Experiment 2 without adjusting outliers. Descriptive statistics are reported in Table S5.1 for both Experiment 1 and Experiment 2.

In Experiment 1, we found a significant main effect of group, *F*(1, 353) = 14.00, *p* < .001, $\eta_{p}^{2}$ = .04, a significant main effect of time, *F*(1, 353) = 14.47, *p* < .001, $\eta_{p}^{2}$ = .04, and a significant interaction effect of group and time, *F*(1, 353) = 41.80 *p* < .001, $\eta_{p}^{2}$ = .11. Simple effect analyses revealed that the misinformed group had a lower attitude towards trusted working hours at t1 compared to the control group, *F*(1, 353) = 24.97, *p* < .001, *d* = 0.53. Further, the groups did differ significantly after the correction was presented, *F*(1, 353) = 4.98, *p* = .026, *d* = 0.24. Moreover, there was a significant increase in attitude from t1 to t2 for the experimental group, *F*(1, 353) = 52.00, *p* < .001, $\eta_{p}^{2}$ = .13, *d_RM_* = 0.49. In the control group, the attitude did not significantly change from t1 to t2, *F*(1, 353) = 3.59, *p* = .059, *d_RM_* = -0.16. Thus, all relevant significances remained the same as in the analyses with adjusted outliers; only the simple effect in the control group, i.e., the decrease in attitude from t1 to t2, was no longer significant.

**Table S5.1**
*Descriptive Statistics Without Adjusting Outliers*

| Group | N | Attitude | | | | Fluid Intelligence | | NFC | |
| --- | --- | --- | --- | --- | --- | --- | --- | --- | --- |
|  |  | t1 | | t2 | |  |  |  |  |
|  |  | *M* | *SD* | *M* | *SD* | *M* | *SD* | *M* | *SD* |
| Experiment 1 |  |  |  |  |  |  |  |  |  |
| Control | 180 | 5.34 | 0.93 | 5.28 | 0.98 | .78 | .17 | 4.56 | 0.91 |
| Experimental | 175 | 4.77 | 1.21 | 5.03 | 1.08 | .77 | .17 | 4.44 | 1.11 |
| Total | 355 | 5.06 | 1.11 | 5.16 | 1.04 | .77 | .17 | 4.50 | 1.01 |
| Experiment 2 |  |  |  |  |  |  |  |  |  |
| Control | 370 | 5.51 | 1.07 | 5.50 | 1.10 | .84 | .16 | 4.85 | 0.93 |
| Experimental | 355 | 5.13 | 1.17 | 5.35 | 1.12 | .84 | .16 | 4.84 | 0.94 |
| Total | 725 | 5.32 | 1.13 | 5.42 | 1.11 | .84 | .16 | 4.85 | 0.93 |

*Note.* Attitude from 1 *(strongly disagree)* to 7 *(totally agree)*, fluid intelligence as the proportion of correct responses, Need for Cognition (NFC) from 1 (*strongly disagree*) to 7 (*strongly agree*).

We calculated a hierarchical regression analysis (see Table S5.2 for results) to test if the correction effect was influenced by fluid intelligence (H3) and by NFC (RQ). The simple slope for the experimental group reflects the effects of fluid intelligence as hypothesized in H3. In support of H3, we observed a significant simple slope of fluid intelligence for the experimental group, *b* = .45, β = .15, *p* = .038, 95% CI [.01, .30], but not for the control group, *b* = -.10, β = -.03, *p* = .634, 95% CI [-.17, .10].

We added NFC to our regression (model 3) to test whether NFC predicts the correction effect, which did not improve the model significantly. Adding the interaction group x NFC (model 4) did also not significantly increase the explained variance in attitude change, and therefore no evidence for NFC influencing the correction effect was found. Neither including the interaction fluid intelligence x NFC (model 5; RQ2) nor the second order interaction group x fluid intelligence x NFC (model 6; RQ3) further improved the regression model. In sum, all tests of hypotheses and tests concerning the research questions revealed the same (non-)significant results as for the analyses using adjusted outliers.

Finally, we calculated a post-hoc regression in which we did not consider the attitude at t1. First, we checked if we found a CIE when controlling for fluid intelligence, i.e., predictors were group and fluid intelligence, attitude at t2 was the criterion. *R^2^* = .02% of the variance in attitude at t2 was explained, *F*(2, 352) = 2.89, *p* = .057, and the group predicted attitude at t2 significantly, *b* = -0.25, *p* = .025. Fluid intelligence was no significant predictor of attitude at t2, *b* = -0.29, β = -0.05, *p* = .371. Next, we added the interaction group x fluid intelligence to the regression analysis. The predictors explained *R^2^* = .02 of variance, *F*(3, 351) = 2.02, *p* = .111. However, the interaction effect was not significant, i.e., variance explained in attitude at t2 did not increase significantly, Δ*R^2^* < .01, *F*(1, 351) = 0.29, *p* = .593. In short, the significances of these analyses remained the same as for the analyses with adjusting outliers.

**Table S5.2**
*Regression of Attitude Change on Experimental Group, Fluid Intelligence, and NFC Without Adjusting Outliers*

| Variable | Model 1 | | Model 2 | | Model 3 | | Model 4 | | Model 5 | | Model 6 | |
| --- | --- | --- | --- | --- | --- | --- | --- | --- | --- | --- | --- | --- |
|  | *b* | *SE* | *b* | *SE* | *b* | *SE* | *b* | *SE* | *b* | *SE* | *b* | *SE* |
| Experiment 1 |  |  |  |  |  |  |  |  |  |  |  |  |
| Group | .33*** | .05 | -.10*** | .24 | -.09*** | .24 | .02*** | .32 | .02*** | .32 | .62*** | 1.06 |
| Fluid intelligence | .16*** | .15 | -.10*** | .21 | -.11*** | .21 | -.12*** | .21 | .57*** | .69 | .99*** | .99 |
| Group x fluid intelligence |  |  | .55*** | .30 | .55*** | .30 | .56*** | .30 | .57*** | .30 | -.21*** | 1.36 |
| NFC |  |  |  |  | .03*** | .03 | .02*** | .03 | .14*** | .12 | .07*** | .16 |
| Group x NFC |  |  |  |  |  |  | .03*** | .05 | .03*** | .05 | .16*** | .24 |
| Fluid intelligence x NFC |  |  |  |  |  |  |  |  | -.16*** | .15 | -.08*** | .20 |
| Group x fluid intelligence x NFC |  |  |  |  |  |  |  |  |  |  | -.18*** | .30 |
| *R^2^* | .11*** |  | .12*** |  | .12*** |  | .12*** |  | .12*** |  | .13*** |  |
| Δ*R^2^* |  |  | .01*** |  | <.01*** |  | < .01*** |  | < .01*** |  | < .01*** |  |
| Experiment 2 |  |  |  |  |  |  |  |  |  |  |  |  |
| Group | .24*** | .03 | -.09*** | .16 | -.09*** | .16 | .01*** | .21 | .02*** | .21 | -.99*** | .77 |
| Fluid intelligence | .21*** | .09 | .02*** | .13 | .03*** | .13 | .02*** | .13 | .92*** | .47 | .38*** | .61 |
| Group x fluid intelligence |  |  | .39*** | .19 | .39*** | .19 | .41*** | .19 | .40*** | .19 | 1.64*** | .93 |
| NFC |  |  |  |  | -.02*** | .02 | -.01*** | .02 | .15*** | .08 | .06*** | .11 |
| Group x NFC |  |  |  |  |  |  | -.02*** | .03 | -.02*** | .03 | .19*** | .16 |
| Fluid intelligence x NFC |  |  |  |  |  |  |  |  | -.19*** | .10 | -.08*** | .13 |
| Group x fluid intelligence x NFC |  |  |  |  |  |  |  |  |  |  | -.26*** | .19 |
| *R^2^* | .09*** |  | .09*** |  | .09*** |  | .09*** |  | .09*** |  | .09*** |  |
| Δ*R^2^* |  |  | .01*** |  | < .01*** |  | < .01*** |  | < .01*** |  | < .01*** |  |

*Note.* Group was dummy-coded using the control group as the reference.
* p < .05, *** p < .001

For Experiment 2, we calculated a 2 (group) x 2 (time) mixed ANOVA (see Table S5.1 for descriptive values) to test the correction effect (H1) and the CIE (H2) using attitude as the dependent variable. Results showed a significant main effect of group, *F*(1, 723) = 10.81, *p* = .001, $\eta_{p}^{2}$ = .02, a significant main effect of the time, *F*(1, 723) = 46.90, *p* < .001, $\eta_{p}^{2}$ = .06, and a significant interaction effect, *F*(1, 723) = 63.73, *p* < .001, $\eta_{p}^{2}$ = .08. Simple effects analysis showed a significant effect of time for the experimental group, *F*(1, 723) = 107.75, *p* < .001, *d_RM_* = 0.48. but not for the control group, *F*(1, 723) = 0.66, *p* = .418, *d_RM_* = -0.05. Therefore, all (non-)significances remained the same as for the analysis using adjusted outliers.

We calculated a hierarchical regression analysis to test if the correction effect was influenced by fluid intelligence (H3) and by NFC (RQ). In model 1, we used group (dummy-coded using the control group as the reference) and fluid intelligence as the predictors and attitude change as the dependent variable. All significances of predictors (except fluid intelligence and NFC in Model 5), total variances explained in attitude change, and changes of variance explained remained the same compared to the analysis with adjusted outliers (see Table S5.1 for descriptive values, Table S5.2 for coefficients and variances explained of all regression models). In support of H3, we observed a significant simple slope of fluid intelligence for the experimental group, *b* = .41, β = .15, *p* = .003, 95% CI [.05, .25], but not for the control group, *b* = .02, β < .01, *p* = .906, 95% CI [-.09, .10].

In Model 5 the increase of variance explained due to the interaction of fluid intelligence and NFC was significant. Therefore, we probed the interaction (fluid intelligence, NFC, and attitude change were standardized for the sake of interpretation). For the experimental group, fluid intelligence had a significant effect on attitude change if NFC was one standard deviation below average, β = .21, *p* < .001, 95% CI [.10, .33], and if NFC was average, β = .15, *p* = .004, 95% CI [.05, .25]. However, the effect of fluid intelligence was no longer significant if NFC was one standard deviation above average, β = .08, *p* = .222, 95% CI [-.05, .21]. For the control group, fluid intelligence did not predict attitude change at any level of NFC (all *p*s > .237). Thus, the significances of the probed slopes remained the same compared to the analysis using adjusted outliers.

As a post-hoc analysis, we regressed the attitude at t2 on fluid intelligence and group without taking the attitude at t2 into account. In total, *R^2^* < .01variance in attitude at t2 was explained, *F*(2, 722) = 1.63, *p* = .198. The results showed no significant main effect of the group, *b* = -0.15, *p* = .072, and no significant main effect of fluid intelligence, *b* = 0.02, *p* = .939. Adding the interaction group x fluid intelligence to the regression model did not increase the variance explained in attitude at t2, Δ*R^2^* < .01, *F*(1, 721) = 1.12, *p* = .291. Therefore, the analyses provided the same significances as the analyses using adjusted outliers.

# S6. Preregistered Regression Analyses NFC

As mentioned in the manuscript, we preregistered to test whether NFC affected the correction effect without taking fluid intelligence into account. Therefore, we calculated a regression analysis using group (dummy coded using the control group as the reference) and NFC as predictors and attitude change as the dependent variable.

For Experiment 1, *R^2^* = .12 variance was explained, *F*(2, 352) = 23.09, *p* < .001. Group predicted attitude change significantly, *b* = 0.34, *p* < .001, however, NFC did not predict attitude change, *b* = 0.03, β = .06, *p* = 278. Next, we added the interaction NFC x group as a predictor. The variance explained did not increase significantly, Δ*R^2^* < 01, *F*(1, 351) = 0.23, *p* = 636. Therefore, NFC did not affect the correction of the attitude in either group replicating the results we obtained when fluid intelligence was included in the regression analysis.

For Experiment 2, *R^2^* = .09 variance was explained, *F*(2, 722) = 35.68, *p* < 001. The group did predict attitude change significantly, *b* = 0.25, *p* < .001, but NFC did not, *b* = -0.02, β = -.03, *p* = .347. Adding the interaction group x NFC as a predictor did not increase the variance explained significantly, Δ*R^2^* < .01, *F*(1, 721) = 0.15, *p* = .704. Thus, NFC did not predict attitude change in any group replicating the results of the hierarchical regression analysis that took fluid intelligence into account.
